# Characterization of Autochthonous Lactic Acid Bacteria Isolated from a Traditional Ethiopian Beverage, *Tella*

**DOI:** 10.3390/foods13040575

**Published:** 2024-02-14

**Authors:** Gashaw Assefa Yehuala, Nurelegne Tefera Shibeshi, Su-Hyeon Kim, Mi-Kyung Park

**Affiliations:** 1 School of Food Science and Biotechnology, Kyungpook National University, Daegu 41566, Republic of Korea; gashawasefa@gmail.com (G.A.Y.); 1sh_hs1@naver.com (S.-H.K.); 2 College of Biological and Chemical Engineering, Addis Ababa Science and Technology University, Addis Ababa P.O. Box 16417, Ethiopia; 3 School of Chemical and Bio-Engineering, Addis Ababa Institute of Technology, Addis Ababa University, Addis Ababa P.O. Box 385, Ethiopia; nutefera@gmail.com; 4 Food and Bio-Industry Institute, Kyungpook National University, Daegu 41566, Republic of Korea

**Keywords:** *Tella*, ethanol tolerance, lactic acid bacteria, functional starter, principal component analysis, probiotic

## Abstract

This study aimed to isolate lactic acid bacteria (LAB) from a traditional Ethiopian fermented product, *Tella*, and evaluate their functional properties. Of forty-three isolates, seven LAB were screened and identified as *Pediococcus pentosaceus*, *Latilactobacillus curvatus*, *Leuconostoc mesenteroides*, and *Lactiplantibacillus plantarum* species. The isolates were tested for their alcohol tolerance, acid and bile resistance, auto-aggregation, co-aggregation, hydrophobicity, antibacterial activity, and antibiotic susceptibility. LAB isolates, specifically *P. pentosaceus* TAA01, *L. mesenteroides* TDB22, and *L. plantarum* TDM41, showed a higher degree of alcohol tolerance in 8% and 10% (*w*/*v*) ethanol concentrations. Additionally, these three isolates displayed survival rates >85% in both acidic pH and bile environments. Among the isolates, *L. plantarum* TDM41 demonstrated the highest auto-aggregation, co-aggregation, and hydrophobicity with (44.9 ± 1.7)%, (41.4 ± 0.2)%, and (52.1 ± 0.1)% values, respectively. The cell-free supernatant of the isolates exhibited antibacterial activity against foodborne pathogens of *Escherichia coli*, *Salmonella* Enteritidis, and *Staphylococcus aureus.* Each isolate exhibited various levels of resistance and susceptibility to seven antibiotics and resistance was observed against four of the antibiotics tested. After performing a principal component analysis, *Pediococcus pentosaceus* TAA01, *L. mesenteroides* TDB22, and *L. plantarum* TDM41 were selected as the most promising ethanol-tolerant probiotic isolates.

## 1. Introduction

Several traditional cereal-fermented beverages are produced in Ethiopia, such as *Tella*, *Borde*, *Cheka*, *Korefe*, *Keribo*, and *Shamita* [1,2]. *Tella* is the most popular fermented alcoholic beverage (2–8%) with an opaque appearance and light yellow to dark brown color [3]. It contains polyphenolic and flavonoid compounds, which offer various biological advantages including protection from free radicals, cancer, and aging [4]. *Tella* is made from various cereals such as barley, maize, wheat, millet, sorghum, and teff, depending on localities and their tradition [5,6].

*Tella* fermentation relies on microorganisms sourced from raw ingredients comprising yeasts (*Saccharomyces*), *Lactobacillus*, *Bacillus*, and other bacteria [3,7]. The traditional *Tella* preparation method usually comprises four phases, namely, the making of “tejet”, “tenses”, “difdif”, and finally *Tella* [3,8]. The starting cereals pass through different preparation methods such as soaking, germinating, roasting, grinding, and baking. The *Tella* preparation process is initiated by soaking barley and germinating it, followed by drying and grinding it into malt flour locally called “bikil” flour. In parallel, “gesho” (*Rhamnus prinoides*) leaves and stems are sun-dried and ponded. Then, “bikil” flour and gesho powder are blended and allowed to ferment for 96 h to create a fermenting mass known as “tejet” [8]. Following this, equal proportions of sorghum, millet, and teff flour are combined with water to produce a dough. The dough is subsequently baked to create unleavened bread, locally known as “kita”, which is then incorporated into the previously prepared “tejet”. The mixture is then sealed tightly and left to ferment for 96 h to turn into “tenses” [8]. While the “tenses” is fermenting, maize grain is soaked in water for about 72 h, and then, it is dried, roasted, and ground to make a dark maize flour called “Asharo”. “Asharo” is then added to the earlier produced “tenses” and fermented for a period of 96 h [8]. After this duration of fermentation, a thick mixture locally known as “difdif” is formed. Water is incorporated into “difdif” and allowed to ferment for a duration of 48 h [3]. Finally, solid residues are filtered out and the resulting liquid is served to consumers as *Tella* [1].

Probiotic bacterial strains contribute to the promotion of good nutrition by aiding in health maintenance through the prevention, control, and treatment of diseases [9,10,11,12]. In *Tella* and similar cereal fermentations, lactic acid bacteria (LAB) play a crucial role in shaping sensory attributes, ensuring safety, and enhancing functionality [7,9,12,13]. LAB are recognized as a key group of probiotic organisms, with numerous strains known for primarily residing in the gastrointestinal tract (GIT) and showing resilience against challenging conditions like low pH, bile salts, natural growth inhibitors, and interactions with other microbes [9]. Functional beverages can be developed through the incorporation of probiotics into food matrices. However, these probiotic microorganisms must exhibit resistance to various stresses encountered during the production process, such as high ethanol content and heat exposure [14].

Dairy products, especially yogurt, have been the common vehicles for probiotics due to the favorable conditions that milk and its derivatives create for these microorganisms [15]. However, there is a significant interest in developing new non-dairy food matrices for probiotic delivery. This trend is driven by a growing consumer preference for plant-based options to address issues like lactose intolerance, cholesterol concerns, and allergic reactions to milk proteins. Consumers highly appreciate food matrices, particularly cereal beverages endowed with functional properties, for their nutritional benefits. Consequently, probiotic-enriched alcoholic beverages have emerged as an innovative solution for delivering beneficial microorganisms. However, navigating the complex alcoholic beverage environment presents challenges in effectively cultivating specific probiotic strains [14].

In the context of probiotic beer development, *Tella* could be the best alternative and readily available source for alcohol-tolerant LAB strains with functional properties, particularly probiotic characteristics. The previous studies of *Tella* were primarily focused on outlining its traditional processing methodologies, physicochemical characteristics, and microbial profiles during fermentation [3,7,16]. Remarkably, there were no studies on the characterizations of LAB isolates such as alcohol tolerance, probiotic properties, and selection of LAB for use as starters in industrial applications. Therefore, the principal objective of this study was isolation and characterization of LAB from *Tella* samples for the selection of potential functional autochthonous starters with better alcohol-tolerant properties. The assessment and selection of the LAB candidates was carried out through a methodical approach encompassing the evaluation of their alcohol tolerance, survival rates and adhesion potential within the GIT, and antibacterial activities. In addition, the overall safety of confirmed LAB isolates was evaluated by an antibiotic susceptibility test to the selected commercial antibiotics.

## 2. Materials and Methods

### 2.1. Tella Samples

Fifteen *Tella* samples were purchased in August from traditional *Tella* breweries in Addis Ababa (AA), Debre Birhan (DB), and Debre Markos (DM), cities in Ethiopia. The capital, Addis Ababa, and the two nearby cities in the northern part of Ethiopia, DB and DM, are well known for the high rate of traditional *Tella* consumption and marketing [7]. Specific *Tella* vendors were selected based on the recommendation of customers to access good-quality *Tella*. Samples were carefully collected using sterile screw glass bottles and transported in an icebox. All samples were kept in a refrigerator until microbial analysis.

### 2.2. LAB Isolation

Lactic acid bacteria isolation was carried out in anaerobic conditions since probiotic strains are required to function in anaerobic conditions during cereal fermentation and in the GIT. Serially diluted *Tella* samples were spread onto De Man–Rogosa–Sharpe (MRS, Kisan Bio Company, Limited (Co., Ltd.), Seoul, Republic of Korea) agar plates supplemented with 50 mg/L cycloheximide (Sigma-Aldrich Co., Saint Louis, MO, USA). After incubation under anaerobic conditions at 30 °C for 48 h, cream to pale yellow colonies displaying circular shapes were randomly selected. These colonies were purified through at least five repetitive streaking cycles on the MRS agar medium. The purified isolates were maintained at −28 °C in MRS broth with 15% glycerol for further phenotypic analyses [17].

### 2.3. Phenotypic Identification of Purified Isolates

The Gram staining, catalase activity, and acidification ability of each LAB isolate were assessed for phenotypic identification. Gram staining was conducted by following the method described in [18]. For the catalase activity of purified single isolates, 3% hydrogen peroxide (Duksan Pure Chemicals Co., Ltd., Ansan, Republic of Korea) was mixed separately on a clean microscope slide with the pure isolates. Positive reactions were evidenced by immediate effervescence (bubble formation) due to the catalase hydrolyzing the hydrogen peroxide [19]. The acidification ability of each purified isolate was assessed by inoculation (6 log CFU/mL) in MRS broth and incubation at 30 °C for 24 h, then measuring its pH [20].

### 2.4. Genotypic Identification of Presumptive LAB Isolates

Presumptive LAB isolates were identified genotypically following the previous method [21]. Briefly, genomic DNA of seven presumptive LAB isolates was extracted and purified using a commercial Genomic DNA Isolation Kit (Solgent Co. Ltd., Daejeon, Republic of Korea). The 16S rRNA gene was amplified with the universal bacterial primer pair 27F and 1492R and the purified PCR products were sequenced by Solgent Co., Daejeon, Republic of Korea. The obtained sequences were analyzed using the basic local alignment search tool (BLAST) and aligned using the multiple alignment software ClustalW algorithm [22]. A phylogenetic tree was constructed using the maximum likelihood method with 100 bootstrap values via MEGA 11 software program [23].

### 2.5. Alcohol Tolerance of LAB Isolates

The alcohol tolerance of each LAB isolate was determined by assessing its ability to withstand challenges posed by ethanol at various concentrations during the fermentation process. The assessments were conducted following established methodologies as described in previous studies [20,24]. The overnight culture of each LAB isolate was individually inoculated at 6 log CFU/mL in MRS broth with varying concentrations of ethanol (2%, 4%, 6%, 8%, and 10%). The measurement of microbial growth was performed after 48 h by reading the absorbance at 600 nm. To serve as controls, samples of unmodified MRS broth inoculated with each LAB isolate were incubated at a temperature of 30°C. For each case, the data were expressed as growth index (GI), a relative measure comparing the growth in an experimental condition (A_s_) relative to the control (A_c_). GI was expressed using the following formula:

GI = A_s_/A_c_ × 100, (1)

### 2.6. Probiotic Characterization of LAB Isolates

#### 2.6.1. Preparation of LAB and Bacterial Culture

The seven confirmed LAB isolates were cultured with MRS broth at 37 °C for 18 h under an anaerobic condition to evaluate their probiotic properties. Pathogenic strains of *Escherichia coli* ATCC 43895, *Salmonella* Enteritidis ATCC 13076, and *Staphylococcus aureus* ATCC 25923 were cultured in tryptic soy broth (TSB, Difco Laboratories Incorporated (Inc.), Sparks, MD, USA) at 37 °C for 18 h. After the specified incubation periods, each LAB isolate and pathogenic bacterial culture was harvested through centrifugation at 6000× *g* for 15 min. The harvested cells were then washed twice with phosphate-buffered saline (PBS, pH 7, Welgene Inc., Gyeongsan, Republic of Korea). Finally, the pellet was resuspended in PBS for further property tests. Cell-free supernatant (CFS) of LAB was prepared by filtering the supernatant obtained post-centrifugation of the overnight LAB culture through a microfilter (0.22 μm, GVS Co. Ltd., Panorama, Los Angeles, CA, USA).

#### 2.6.2. Acid Resistance

The acid resistance of each LAB isolate was evaluated according to the method described by Vijayalakshmi et al. [25], with minor modifications. Each LAB culture was adjusted to pH 3.0 and incubated at 37 °C for 3 h. Sample aliquots were taken at time 0 and after 3 h of incubation, plated on MRS agar plates, and incubated anaerobically at 37 °C for 48 h to determine the survival rate after exposure to low pH. Cultures of each LAB isolate adjusted to pH 7.2 were used as controls. The survival rate was calculated as follows:

(2) $$\mathrm{Surivival}\left(\%\right)=\frac{\mathrm{Number}\;\mathrm{of}\;surviving\;cells\;after\;3\;h\;of\;incubation\;(\log\;CFU/mL)}{Ininital\;number\;of\;cells\;before\;incubation\;(\log\;CFU/mL)}\times 100,$$

#### 2.6.3. Bile Salt Tolerance

Bile salt tolerance of each LAB isolate was evaluated based on the methods described by Mallappa et al. [26], with minor modifications. Briefly, 1% LAB culture was inoculated in MRS broth supplemented with 0.3% (*w*/*v*) bile salt (Sigma-Aldrich Co., Ltd., USA) and incubated anaerobically at 37 °C for 4 h. Sample aliquots were taken at time 0 and after 4 h of incubation, plated on MRS agar plates, and incubated anaerobically at 37 °C for 48 h to determine the survival rate after exposure to bile salts. Cultures of each LAB isolate without bile salt were used as controls. The survival rate was calculated as follows:

(3) $$Surivival\left(\%\right)=\frac{Number\;of\;surviving\;ceslls\;after\;4\;h\;of\;incubation\;\log(CFU/mL)}{Ininital\;number\;of\;cells\;prior\;to\;incubation\;(logCFU/mL)}\times 100,$$

#### 2.6.4. Cell Auto-Aggregation

Auto-aggregation of each LAB isolate was determined following the method described by Mallappa et al. [26]. The absorbance (A_0_) of each LAB culture was adjusted with PBS to approximately 0.8 at 600 nm. After incubation at 37 °C for 4 h, the absorbance (A_t_) of the upper fraction of incubated culture suspension was measured and the percentage of auto-aggregation was determined as follows:

Cell auto-aggregation (%) = (1 − A_t_/A_0_) × 100, (4)

#### 2.6.5. Co-Aggregation

The co-aggregation property of each LAB isolate with the pathogenic strains was assessed following the method outlined by Mallappa et al. [26]. The initial absorbances of each LAB culture (A_lac_) and pathogenic bacterial culture (A_path_) were adjusted to 0.8 ± 0.05 (8 log CFU/mL) and 0.3 ± 0.05 (8 log CFU/mL) at 600 nm, respectively. Then, equal volumes (1.5 mL) of each LAB culture and pathogenic bacterial culture were vortexed for 10 sec prior to incubation. After 4 h incubation at 37 °C, the absorbance of the mixture (A_mix_) was measured to calculate the co-aggregation rate:

Co-aggregation (%) = [((A_lac_ + A_path_)/2 − A_mix_)/(A_lac_ + A_path_)/2] × 100, (5)

#### 2.6.6. Cell Surface Hydrophobicity

Each isolate’s hydrophobicity was determined by its adhesion ability to organic solvents, based on the procedure described by Muñoz-Provencio et al. [27], with some modifications. Each LAB culture was adjusted to 8 log CFU/mL and the initial absorbance at 600 nm was measured (A_0_). A total of 3 mL of each LAB culture was separately mixed with 1 mL of xylene (Samchun Pure Chemical Co., Ltd., Pyeongtaek, Republic of Korea) and chloroform (Duksan Pure Chemicals Co., Ltd., Ansan-si, Republic of Korea). The mixtures were incubated at 37 °C for 10 min, vortexed thoroughly, and incubated again at 37 °C for 4 h. After incubation, the aqueous phase was removed, and the absorbance (A_t_) was measured at 600 nm for the calculation of cell surface hydrophobicity using the following formula:

Cell surface hydrophobicity (%) = (1 − A_t_/A_0_) × 100, (6)

### 2.7. Antibiotic Susceptibility

The disc diffusion method [28,29] was employed to assess the antibiotic susceptibility of LAB isolates using eleven commercial antibiotics (Oxoid Ltd., Basingstoke, UK), including ampicillin, gentamicin, kanamycin, streptomycin, erythromycin, tetracycline, chloramphenicol, penicillin G, trimethoprim/sulfamethoxazole, ciprofloxacin, and azithromycin. A total of 100 μL of each LAB isolate suspension (8 log CFU/mL) was evenly spread on an MRS agar plate prior to the placement of each antibiotic disc. The antibiotic concentration on the discs is specified in Table S1. After anaerobic incubation at 37 °C for 24 h, each inhibition zone was measured for the determination of its susceptibility following the Clinical and Laboratory Standards [30] criteria.

### 2.8. Antibacterial Activity

Each LAB isolate’s antibacterial activity was tested against three pathogens, *E. coli* ATCC 43895, *S. Enteritidis* ATCC 13076, and *S. aureus* ATCC 25923, using the well diffusion method described by Sakoui et al. [31]. Briefly, 1% (*v*/*v*) of each pathogenic bacterial culture (8 log CFU/mL) was inoculated separately into molten Luria–Bertani agar (Difco, Sparks, MD, USA). After solidification, wells of 7 mm in diameter were prepared, and 100 μL of CFS was added to each well. Following diffusion for 4 h at 4 °C, the plates were incubated at 37 °C for 24 h. As a control, fresh MRS broth was utilized instead of CFS. The diameter (mm) of the clear zone around the wells was measured to compare antibacterial activity.

### 2.9. Statistical Analysis

All of the tests were performed in three independent experiments. The experimental results were expressed as mean ± standard deviation. The data were subjected to a one-way analysis of variance using GraphPad Prism 8.3.0 (GraphPad Software Inc., La Jolla, CA, USA) with *p* < 0.05 for statistical significance. Probiotic properties, including resistance to low pH and bile salts, auto-aggregation and co-aggregation, hydrophobicity, and antibacterial activity, were subjected to a principal component analysis (PCA) using Minitab 19.2 Statistical Software (Minitab Inc., State College, PA, USA) to select the best LAB isolates.

## 3. Results and Discussion 

### 3.1. Isolation and Phenotypic Characterization of Presumptive LAB Isolates

Based on disparities in macroscopic characteristics, 43 bacterial colonies were isolated from 15 *Tella* samples (Table 1). Out of the 43 initial colonies, 33 were Gram-positive, 19 were catalase-negative, and 15 were both Gram-positive and catalase-negative. The cell morphology of these fifteen isolates consisted of four cocci, nine bacilli, one streptococcus, and one streptobacillus (Table 2). Based on the criteria defined by Amelia et al. [32], LAB encompass Gram-positive and catalase-negative bacteria. Consequently, 15 isolates with both characteristics were selected for further physiological tests, specifically evaluating their acidification ability, as detailed in Table 2. Seven isolates, including TAA01, TAA04, TDB19, TDB21, TDB22, TDM40, and TDM41, were able to reduce the pH of the MRS broth by >1.0. Therefore, these isolates were considered presumptive LAB with potential application for *Tella* fermentation and were subjected to genotypic identification.

### 3.2. Genotypic Identification

**Figure 1:**
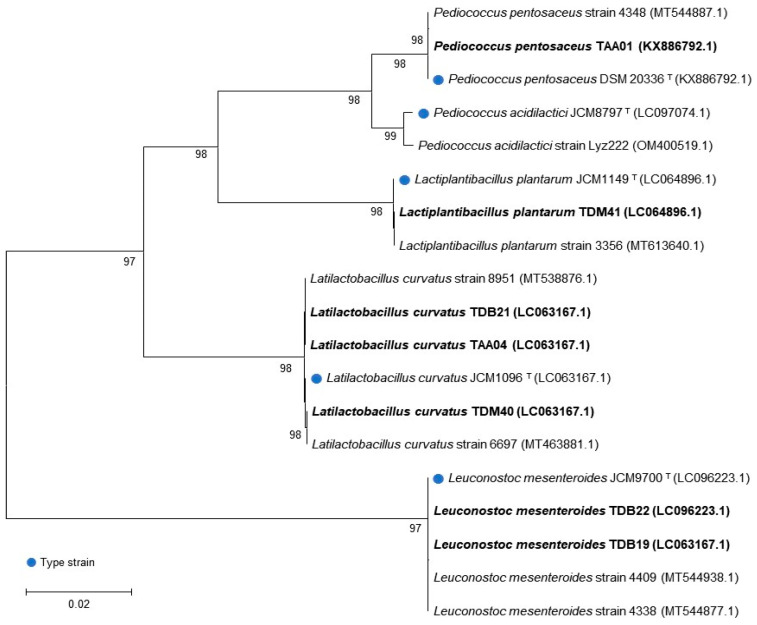
Phylogenic analysis of autochthonous lactic acid bacteria isolates based on 16S rRNA gene sequencing. The number next to the branches indicates the percentage of replicate trees in which the associated taxa clustered together in the bootstrap test with 100 replicates.

The genetic analysis of the presumptive LAB isolates identified the following genera: *Pediococcus* (one isolate), *Latilactobacillus* (three isolates), *Leuconostoc* (two isolates), and *Lactiplantibacillus* (one isolate) (Table 2). The BLAST hit displaying the maximum identity and query length was used as the closest relative of the isolates whose sequences were acquired. All isolates exhibited a similarity of >99% with the nucleotide sequence database of the National Center for Biotechnology Information (NCBI). The genetic sequence of each isolate was deposited in the NCBI GenBank database, and accession numbers were obtained. The identification and classification of the isolates were further confirmed by phylogenetic tree analysis (Figure 1), where each LAB isolate (written in bold) was classified with type strains (indicated by blue dots and “T” superscripts) and other similar strains in the database.

*Pediococcus pentosaceus* was isolated from AA *Tella* samples, two *Leuconostoc mesenteroides* were isolated from DB samples, and *Lactiplantibacillus plantarum* isolate was isolated from DM. On the other hand, *Latilactobacillus curvatus* isolates were consistently present in *Tella* samples collected from all sites. LAB’s predominant presence in *Tella* has been previously reported [3,7], although it is important to note that variations in seasonal conditions and processing practices may contribute to differences in the identified species of isolates. Following identification, all seven isolates underwent a series of tests specifically designed to evaluate their alcohol tolerance and probiotic characteristics. This comprehensive evaluation could be used to identify the most promising functional isolates for *Tella* fermentation and other functional food industry applications within the group.

### 3.3. Alcohol Tolerance Ability

**Figure 2:**
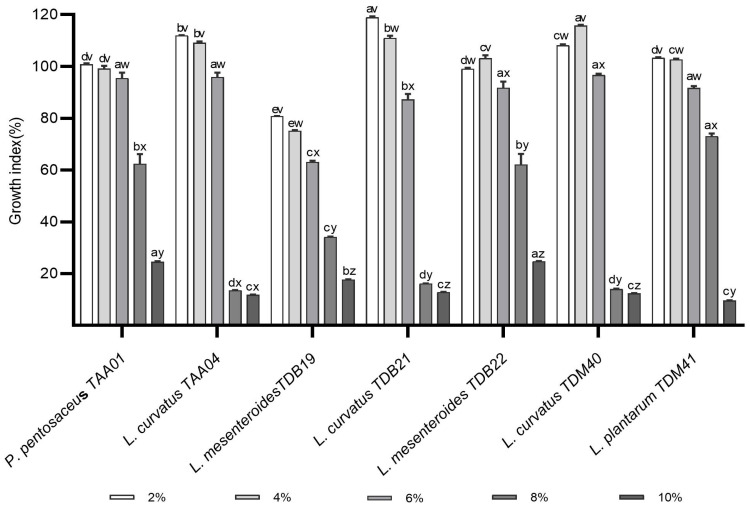
Alcohol tolerance abilities of autochthonous lactic acid bacteria isolates exposed to 2%, 4%, 6%, 8%, and 10% ethanol (*v*/*v*) in MRS broth. Values are expressed as mean ± SD (*n* = 3). Different superscript letters (a–e) represent significant differences (*p* < 0.05) between different isolates under the same treatment and (v–z) represent significant differences within the same isolates across different treatments.

The alcohol content of *Tella* varies between 2% and 8% *v*/*v* [3]. High alcohol concentration affects both the growth and activity of LAB. When the concentration of ethanol surpasses a specific threshold, LAB are unable to autonomously sustain cellular stability, potentially leading to cell death. This outcome is attributed to intracellular metabolic imbalance resulting from the disruption of cell membrane functions [33]. Therefore, starter LAB strains to be employed in *Tella* fermentation as well as other food fermentations should have better resistance to alcohol stresses. In the present study, the alcohol tolerance of each LAB isolate was determined for ethanol concentrations ranging from 2 to 10% (Figure 2). As shown in Figure 2, there is a decreasing trend of GI of LAB isolates with the increase in ethanol concentration. The average GI was 104.2 ± 13.5%, 102.3 ± 13.2%, 88.9 ± 12.1%, 39.4 ± 26.0%, and 16.3 ± 6.2% for ethanol concentrations of 2%, 4%, 6%, 8%, and 10%, respectively. Notably, the GI values for certain isolates at 2% and 4% exceeded those of the control, indicating a potential promotion of their growth at lower alcohol concentrations. Such growth promotion at the low alcohol concentration may be due to the adaptation of the isolates to alcoholic conditions, where the LAB isolate may have developed different ethanol tolerance mechanisms. Among the isolates, *P. pentosaceus* TAA01 and *L. curvatus* TAA04 were not significantly inhibited by the increase in ethanol from 2% to 4%. At a 6% ethanol concentration, the GI of LAB isolates varied in the following order from highest to lowest: *L. curvatus* TDM40 (96.7 ± 0.5)%, *L. curvatus* TAA04 (95.9 ± 1.7)%, *P. pentosaceus* TAA01 (95.5 ± 2.1)%, *L. mesenteroides* TDB22 (91.8 ± 2.4)%, *L. plantarum* TDM41 (91.7 ± 0.7)%, *L. curvatus* TDB21 (87.3 ± 2.0)%, and *L. mesenteroides* TDB19 (63.1 ± 0.5)%. In this ranking, there is no significant GI difference (*p* > 0.05) among the top five isolates (Figure 2). As the ethanol concentration rose to 8% and 10%, the tolerance capabilities of LAB isolates exhibited more pronounced variations. *P. pentosaceus* TAA01, *L. mesenteroides* TDB22, and *L. plantarum* TDM41 were the most tolerant in 8% ethanol, and *P. pentosaceus* TAA01, *L. mesenteroides* TDB19, and *L. mesenteroides* TDB22 were the most tolerant at a 10% ethanol concentration. These findings closely agree with the study of [33,34]. Another study by Jin et al. [35] reported that *L. paracasei* isolates displayed a 5% alcohol tolerance. Compared to these previous studies, three isolates in the present study including *P. pentosaceus* TAA01, *L. mesenteroides* TDB22, and *L. plantarum* TDM41 exhibited a better GI (above 62%) under an 8% alcohol level. Remarkably, when subjected to the 8% ethanol concentration, *L. plantarum* TDM41 demonstrated a better GI of 73.08%, surpassing the earlier reported GI value of *L. plantarum* LTJ12 (59.01%) by Wang et al. [34]. Isolates of LAB with better alcohol tolerance have the potential to improve fermentation efficiency and stay viable during storage. This is particularly significant because such tolerant LAB strains could be incorporated into products delivered to consumers, ensuring viable probiotic counts and delivering health benefits. The findings of the current study, particularly those regarding the top three alcohol-tolerant isolates, indicate promising opportunities for developing functional cereal-based alcoholic beverages that cater to consumers seeking non-dairy options. However, further studies using *Tella* fermentation models are required to establish more robust conclusions.

### 3.4. Probiotic Properties

“Probiotics are living microorganisms that impart benefits to the host when administered in adequate quantities” [36]. They are essential in preparing functional beverages. Commonly, probiotics have been selected for their stress-resistant phenotypes, ensuring their survival through the GIT and subsequent establishment in the gut. However, the biological properties responsible for their health-promoting effects (e.g., antioxidant activities) and safety characteristics (e.g., antibiotic susceptibility) remain too important [37]. This study identified functional isolates among the screened LAB and evaluated their probiotic properties, including resistance to low pH and bile salts, auto-aggregation and co-aggregation, hydrophobicity, antibacterial activity, and antibiotic susceptibility.

#### 3.4.1. Resistance to Low pH and Bile Salts

Acid and bile salt resistance are among the crucial criteria used to select isolates with probiotic functionality. Acid tolerance is essential to withstand the unfavorable conditions of the GIT and enables the strain to survive longer in high-acid beverages, such as yogurt and *Tella* [7,38]. To thrive and colonize in the human GIT successfully, probiotics ideally should possess traits that enable them to withstand the acidity of the stomach and endure exposure to bile in the upper portion of the intestine [26,31]. The majority of in vitro assays have been designed to identify strains capable of enduring the harsh conditions of the gastrointestinal tract, such as low pH values ranging from 2.0 to 3.0 and bile concentrations of 0.3% [26,39,40]. In this study, LAB isolates underwent evaluation in conditions reflective of the stomach environment, encompassing a pH of 3.0, a 0.3% bile salt concentration, and an exposure time of 3 h. However, it is worth checking the survival ability of the isolates at various pH levels in the range to ensure their versatile application.

**Figure 3:**
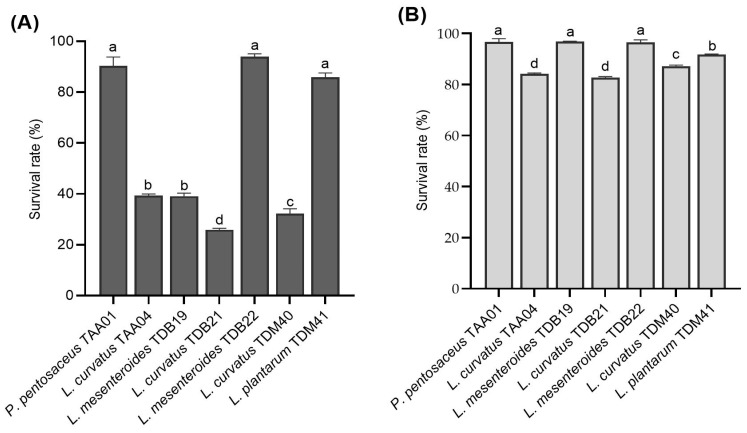
Survival rates of autochthonous lactic acid bacteria isolates following exposure to (**A**) low pH (3.0) for 3 h and (**B**) bile salt (0.3%) for 4 h (count, log CFU/mL). Values are expressed as mean ± SD (*n* = 3). Different superscript letters (a–d) indicate a significant difference (*p* < 0.05).

The LAB isolates displayed various resistance levels to low pH (3.0) and bile salt concentration (0.3%) ranging between (25.93 ± 0.6)% and (94.03 ± 1.1)% and between (82.71 ± 0.4)% and (96.92 ± 0.1)%, respectively (Figure 3A,B). The control samples in both acid and bile salt tolerance experiments did not show significant viability changes between the initial and final counts. Isolates *P. pentosaceus* TAA01, *L. mesenteroides* TDB22, and *L. plantarum* TDM41 displayed the highest resistance rates to low pH, with survival rates of (90.33 ± 3.52)%, (94.03 ± 1.06)%, and (85.93 ± 1.64)%, respectively. Regarding bile salt conditions, *P. pentosaceus* TAA01, *L. mesenteroides* TDB19, and *L. mesenteroides* TDB22 displayed the highest resistance, with survival rates of (96.75 ± 1.26)%, (96.92 ± 0.09)%, and (96.47 ± 1.07)%, respectively. The results are in close agreement with previous studies [20,26,41]. Notably, similar species, including *L. mesenteroides* (TDB19 and TDB22) and *L. curvatus* (TAA04, TDB21, and TDM40), displayed significantly different (*p* < 0.05) survival rates for both low pH and bile salt resistance, underscoring the crucial factor of strain specificity in the selection of probiotic strains [9,42]. TDB19 and TDB22, both belonging to *L. mesenteroides*, exhibited differences in survival rates, with TDB22 demonstrating superior survival in both acid and bile salt treatments. Similarly, within the *L. curvatus species*, TAA04, TDB21, and TDM40 displayed distinct acid resistance abilities, with TDM40 showing enhanced bile tolerance compared to TAA04 and TDB21. Isolates displaying strong acid resistance, like *P. pentosaceus* TAA01, *L. mesenteroides* TDB22, and *L. plantarum* TDM41, indicate their ability to remain viable in acidic environments, both during fermentation and exposure to gastrointestinal fluids. Using these isolates as starter cultures for fermenting cereal beverages, such as *Tella*, offers an exciting opportunity for effectively delivering probiotics. However, evaluating the survival capacity of these isolates in vivo remains prudent.

#### 3.4.2. Auto-Aggregation and Co-Aggregation Properties

The probiotic ability to self-aggregate and co-aggregate with pathogenic bacteria is a good indicator of gut colonization [43]. The aggregation ability of probiotics is related to their cell adherence properties and specifies their capability to survive and persist in the GIT, beneficially affecting their host [9]. Among the tested LAB isolates, *L. plantarum* TDM41 displayed the highest auto-aggregation ability (44.9 ± 1.7)% and *L. mesenteroides* TDB19 the lowest (24.5 ± 1.9%) (Table 3). The auto-aggregation properties of the LAB isolates in the current study displayed higher values than those reported by Vijayalakshmi et al. [25] and lower values than those of Sakoui et al. [31]. Additionally, similar isolates of *L. mesenteroides* (TDB19 and TDB22) displayed significantly different auto-aggregation properties, indicating that auto-aggregation properties are also strain-specific [26].

A co-aggregation assay between the LAB isolates and pathogenic strains, including *E. coli*, *S. Enteritidis*, and *S. aureus*, was performed to evaluate interbacterial adherence. The LAB isolates displayed co-aggregation values between (19.5 ± 0.3)% (*L. mesenteroides* TDB19 with *E. coli* ATCC 43895) and (41.4 ± 0.2)% (*L. plantarum* TDM41 with *S. aureus* ATCC 25923) (Table 3). Wider ranges (7.0–70.0)% of LAB co-aggregation properties with other pathogens, including *E. coli* ATCC8539, *L. monocytogenes* ATCC19115, *S. aureus* ATCC 114, and *S.* Typhimurium LT2, have been previously reported [26,31,44], indicating that the co-aggregation properties in this study were moderate. Furthermore, our findings indicate that LAB co-aggregation with pathogens and their ability to adhere to the epithelial cell surface are strain-specific [26]. This specificity was demonstrated by the significantly different (*p* < 0.05) co-aggregation ability of *L. curvatus* isolates to *E. coli* ATCC 43895, *L. mesenteroides* isolates to *S. Enteritidis* ATCC 13076, and *L. mesenteroides* isolates to *S. aureus* ATCC 25923. Such variability can be explained by the presence of specific molecules on the surface of the LAB isolates acting as ligands for pathogen binding [45]. The co-aggregation of probiotics with pathogenic bacteria could also form a defensive barrier that may inhibit pathogens from colonizing the gut [46]. Good co-aggregation properties, along with auto-aggregation, can be used as a guide for selecting probiotic strains [47,48].

#### 3.4.3. Hydrophobicity

Cell surface hydrophobicity is an essential probiotic property that influences bacterial adhesion and interaction with the host cells in the GIT [49,50]. Here, all isolates were evaluated for their ability to adhere to two organic solvents: chloroform and xylene. The isolates’ hydrophobicity ranged from (17.0 ± 0.4)% in xylene to (52.1 ± 0.1)% in chloroform (Table 3). The hydrophobicity of LAB isolates was generally higher in chloroform than in xylene. *L. plantarum* TDM41 displayed the highest hydrophobicity toward xylene (45.4 ± 0.1)% and chloroform (52.1 ± 0.1)%. On the other hand, *L. mesenteroides* TDB19 displayed the lowest values toward xylene (17.0% ± 0.4%) and chloroform (18.0 ± 0.1)%. Many studies have reported a hydrophobicity ranging from (2.0 ± 0.2)% to (88.0 ± 0.2)% [9,26,31,51]. Compared to such studies, our LAB isolates had a moderate hydrophobicity, indicating their ability to competitively attach to the epithelial cells and promote health [52].

### 3.5. Antibiotic Susceptibility

The food matrix can influence the spread of antimicrobial resistance by providing a suitable environment for the survival of resistant and multiresistant bacteria [53]. The proliferation of bacteria resistant to antimicrobial agents poses a significant threat to human health [54]. Therefore, evaluating antimicrobial resistance in indigenous LAB isolates is a crucial safety measure, given that fermented foods can disseminate these resistant microorganisms. In addition, horizontal gene transfer of antibiotic-resistant genes from probiotics to pathogenic bacteria is possible; however, LAB have been “generally recognized as safe” [51]. In this study, all seven LAB isolates were sensitive to erythromycin, tetracycline, chloramphenicol, and azithromycin but resistant to ampicillin, gentamycin, kanamycin, and streptomycin (Table 4). Gentamycin, kanamycin, and streptomycin are antibiotics that generally target Gram-negative bacteria. Due to their thick cell wall, Gram-positive LAB are resistant to these antibiotics [55]. In addition, resistance to aminoglycosides, including gentamicin, kanamycin, and streptomycin, is intrinsic to the *Lactobacillus* genus and cannot present safety problems [56,57]. Most LAB isolates displayed intermediate susceptibility to penicillin, while the three *L. curvatus* isolates were susceptible. All isolates displayed resistance to trimethoprim/sulfamethoxazole, except *L. plantarum* TDM41. Furthermore, four isolates displayed resistance to ciprofloxacin, while the other three exhibited intermediate susceptibility. The findings align with prior research, indicating that LAB isolates exhibit sensitivity to antibiotics that impede protein synthesis, such as chloramphenicol, erythromycin, and tetracycline. Conversely, resistance was observed against aminoglycosides, including gentamycin, kanamycin, and streptomycin [9,58]. The different susceptibility levels of the LAB isolates toward penicillin and ciprofloxacin indicate strain specificity [59]. Probiotic isolates should be susceptible to at least two clinically relevant antibiotics [60]; therefore, the isolates in this study meet the safety requirements in terms of phenotypic resistance evaluation. Additionally, isolates can harbor truncated antimicrobial resistance genes without expressing the phenotypic resistance pattern [61] or they can show phenotypic resistance to multiple antimicrobial compounds without presenting commonly evaluated resistance genes. Hence, it is imperative to examine the key resistance genes even when an isolate does not exhibit phenotypic resistance. If isolates carry any resistance gene, it becomes crucial to assess both the expression of that gene and the existence of mobile genetic elements capable of transferring these resistance determinants to other bacteria [62].

### 3.6. Antibacterial Activity

The antibacterial activity of LAB is essential for their selection as candidates for starter culture development and probiotic application [63,64,65]. Selecting a starter culture candidate with better antagonistic properties toward harmful pathogens in the fermentation medium and GIT could help produce improved quality products: the so-called functional beverages. The antibacterial activity levels of the CFS against test pathogens, including *E. coli* ATCC 43895, *S. Enteritidis* ATCC 13076, and *S. aureus* ATCC 25923, are presented in Table 5. The CFS from all LAB isolates displayed antibacterial activity against all of the test pathogens, except the non-detectable effect of *L. curvatus* TAA04 and TDM40 on *S. aureus* ATCC 25923 and *L. mesenteroides* TDB19 on *E. coli* ATCC 43895 and *S. Enteritidis* ATCC 13076. *E. coli* ATCC 43895 and *S. Enteritidis* ATCC 13076 were inhibited by six LAB isolates, whereas *S. aureus* ATCC 25923 was inhibited by five. The highest inhibition zone (17.0 ± 1 mm) was observed on *S. Enteritidis* ATCC 13076 by *L. mesenteroides* TDB22, and the lowest (9.0 ± 1.0 mm) was on *S. Enteritidis* ATCC 13076 by *L. curvatus* TDM40. Overall, *P. pentosaceus* TAA01, *L. mesenteroides* TDB22, and *L. plantarum* TDM41 exhibited better antibacterial activity than other isolates. Similar findings have been reported in several studies [31,43]. The production of organic acids, hydrogen peroxide, and bacteriocins by LAB is responsible for the inhibition effect on pathogens [63,65,66]. In general, the results demonstrated that the LAB isolates had a more effective antibacterial activity against *E. coli* ATCC 43895 and *S. Enteritidis* ATCC 13076 than against *S. aureus* ATCC 25923.

### 3.7. Functional Starter Selection

**Figure 4:**
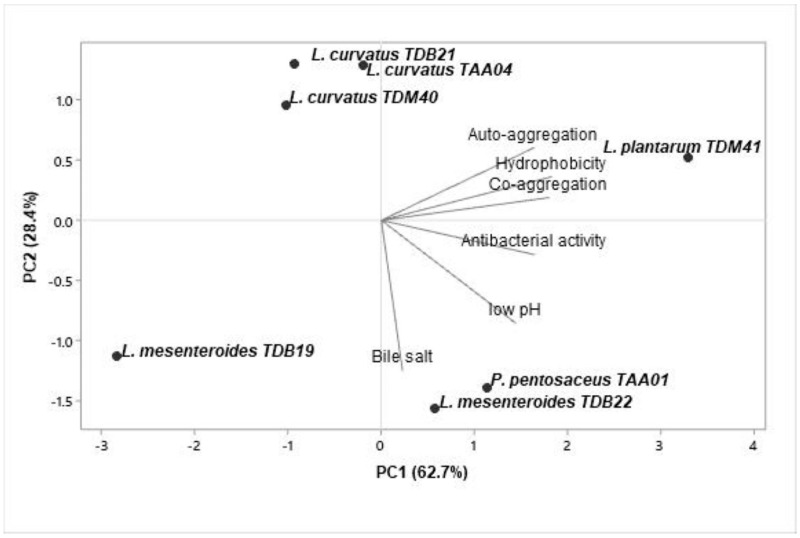
Principal component analysis of probiotic properties (low pH resistance, bile salt resistance, auto-aggregation, co-aggregation, cell surface hydrophobicity, and antibacterial activity) of seven autochthonous lactic acid bacteria isolates. The graph is a biplot displaying the projection of variables and LAB isolates formed by the two major principal components.

The obtained data were subjected to a multivariate PCA using Minitab 19.2 software to evaluate the similarity and variability between the functional properties of the isolates and select potential starter candidates. The analyzed data include acid and bile salt resistance, auto-aggregation and co-aggregation, cell surface hydrophobicity, and antimicrobial activity. The first two PCs accounted for 91.1% of the total variance (Figure 4). PC1 and PC2 contributed 62.7% and 28.4%, respectively. The biplot based on PC1 and PC2 differentiated the isolates into four quadrants. Isolates in quadrants II and IV displayed a better correlation to PC1, indicating that *P. pentosaceus* TAA01, *L. mesenteroides* TDB22, and *L. plantarum* TDM41 are good candidates for potential industrial applications.

## 4. Conclusions

It is undeniable that probiotic microbes are incredibly important for our health and well-being. The LAB isolated from *Tella*, especially *P. pentosaceus* TAA01, *L. mesenteroides* TDB22, and *L. plantarum* TDM41, showed remarkable resilience, tolerating ethanol concentrations up to 8%. These isolates also demonstrated superior survival under conditions simulating the GIT. Moreover, all of the isolated strains displayed moderate to high adhesion properties, with *L. plantarum* TDM41 standing out as the top performer in terms of auto-aggregation, co-aggregation, and hydrophobicity. While susceptibility to several antibiotics was observed in all strains, they also exhibited antimicrobial potential. The present study’s findings highlight the effectiveness of PCA in selecting the most promising probiotic isolates, with *P. pentosaceus* TAA01, *L. mesenteroides* TDB22, and *L. plantarum* TDM41 showing promising functional properties among the LAB isolates. However, there is a need for further exploration, particularly in establishing *Tella* model systems to assess technological properties, the impact on organoleptic qualities, and in vivo investigation of the probiotic properties. This additional evaluation will pave the way for implementing an indigenous starter culture in *Tella* fermentation, contributing to the development of functional beverages.

## Figures and Tables

**Table 1:** Isolation and distribution of presumptive lactic acid bacteria (LAB) in *Tella* samples from three different areas of Ethiopia.

Collection Cites	No. of LAB -Positive Samples	No. of Single Isolates	Isolate Code ^1^	Physiological Test		No. of Presumptive LAB Isolates ^2^
				No. of Gram-Positive Isolates	No. of Catalase -Negative Isolates	
Addis Ababa (*n* = 5)	5	16	TAA01–TAA16	13	6	3
Debre Birhan (*n* = 5)	3	14	TDB17–TDB30	9	6	6
Debre Markos (*n* = 5)	5	13	TDM31–TDM43	11	7	6

^1^ T, *Tella*; AA, Addis Ababa; DB, Debre Birhan; DM, Debre Markos. ^2^ Presumptive LAB isolates are Gram-positive and catalase-negative isolates.

**Table 2:** Phenotypic characterization of presumptive LAB isolates and their genotypic identification using 16S rRNA sequencing.

Isolate Code	Cell Morphology	Acidification (ΔpH > 1) ^1^	Genotype Identification			
			The Closest Type Strain	Query Length (bp)	Identity (%)	Accession Number
TAA01	Coccus	+	*Pediococcus pentosaceus* DSM 20336 ^T^	1441	100.0	KX886792.1
TAA04	Bacillus	+	*Lactiplantibacillus curvatus* JCM 1096 ^T^	1440	100.0	LC063167.1
TAA14	Bacillus	−	ND	ND	ND	ND
TDB18	Streptococcus	−	ND	ND	ND	ND
TDB19	Coccus	+	*Leuconostoc mesenteroides* JCM 9700 ^T^	1418	100.0	LC063167.1
TDB21	Bacillus	+	*Lactiplantibacillus curvatus* JCM 1096 ^T^	1435	99.9	LC063167.1
TDB22	Coccus	+	*Leuconostoc mesenteroides* JCM 9700 ^T^	1417	100.0	LC096223.1
TDB23	Coccus	−	ND	ND	ND	ND
TDB24	Bacillus	−	ND	ND	ND	ND
TDM32	Bacillus	−	ND	ND	ND	ND
TDM34	Streptobacillus	−	ND	ND	ND	ND
TDM35	Bacillus	−	ND	ND	ND	ND
TDM38	Bacillus	−	ND	ND	ND	ND
TDM40	Bacillus	+	*Lactiplantibacillus curvatus* JCM 1096 ^T^	1440	100.0	LC063167.1
TDM41	Bacillus	+	*Lactiplantibacillus plantarum* JCM 1149 ^T^	1438	100.0	LC064896.1

^1^ Acidification ability (ΔpH): the difference in pH was recorded before and after inoculation with individual isolates and incubation at 30 °C for 4 h. T, type strain. +, ΔpH > 1. −, ΔpH < 1. ND, not determined.

**Table 3:** Auto-aggregation, hydrophobicity, and co-aggregation properties of autochthonous lactic acid bacteria (LAB) isolates.

LAB Isolates	Auto- Aggregation (%)	Co-Aggregation (%)			Hydrophobicity (%)	
		*E. coli* ATCC 43895	*S. Enteritidis* ATCC 13076	*S. aureus* ATCC 25923	Xylene	Chloroform
*P. pentosaceus* TAA01	31.7 ± 0.1 ^b^	23.3 ± 0.1 ^c^	29.4 ± 0.8 ^a^	32.0 ± 0.1 ^b^	32.2 ± 0.6 ^c^	35.3 ± 0.3 ^c^
*L. curvatus* TAA04	33.4 ± 1.1 ^b^	26.5 ± 0.4 ^b^	26.6 ± 0.1 ^b^	27.0 ± 0.4 ^c^	31.7 ± 0.6 ^c^	39.8 ± 0.6 ^b^
*L. mesenteroides* TDB19	24.5 ± 1.9 ^c^	19.5 ± 0.3 ^f^	20.7 ± 0.2 ^d^	21.6 ± 0.1 ^e^	17.0 ± 0.4 ^e^	18.0 ± 0.1 ^g^
*L. curvatus* TDB21	33.8 ± 1.4 ^b^	23.8 ± 0.2 ^c^	21.2 ± 0.1 ^d^	24.2 ± 0.4 ^d^	28.1 ± 0.9 ^d^	25.9 ± 0.6 ^f^
*L. mesenteroides* TDB22	32.3 ± 0.5 ^b^	20.5 ± 0.5 ^e^	29.9 ± 0.8 ^a^	20.2 ± 0.1 ^f^	34.5 ± 0.2 ^b^	28.1 ± 0.1 ^e^
*L. curvatus* TDM40	34.5 ± 1.8 ^b^	21.9 ± 0.4 ^d^	23.2 ± 0.4 ^c^	24.0 ± 0.4 ^d^	29.2 ± 1.2 ^d^	31.6 ± 0.6 ^d^
*L. plantarum* TDM41	44.9 ± 1.7 ^a^	34.0 ± 0.5 ^a^	23.5 ± 0.5 ^c^	41.4 ± 0.2 ^a^	45.4 ± 0.1 ^a^	52.1 ± 0.1 ^a^

Data represent the mean ± standard deviation of three independent experiments. Different superscript letters (a–g) represent significant differences (*p* < 0.05) among the means within the same column.

**Table 4:** Antibiotic susceptibilities of autochthonous lactic acid bacteria (LAB) isolates.

LAB Isolates	Antibiotics ^1^										
	AMP	GEN	KAN	STR	ERY	TET	CHL	PEN	SXT	CIP	AZM
*P. pentosaceus* TAA01	R	R	R	R	S	S	S	I	R	R	S
*L. curvatus* TAA04	R	R	R	R	S	S	S	S	R	I	S
*L. mesenteroides* TDB19	R	R	R	R	S	S	S	I	R	I	S
*L. curvatus* TDB21	R	R	R	R	S	S	S	S	R	R	S
*L. mesenteroides* TDB22	R	R	R	R	S	S	S	I	R	R	S
*L. curvatus* TDM40	R	R	R	R	S	S	S	S	R	I	S
*L. plantarum* TDM41	R	R	R	R	S	S	S	I	S	R	S

^1^ AMP, ampicillin (10 µg); GEN, gentamicin (10 µg); KAN, kanamycin (30 µg); STR, streptomycin (10 µg); ERY, erythromycin (15 µg); TET, tetracycline (30 µg); CHL, chloramphenicol (30 µg); PEN, penicillin G (10 µg); SXT, trimethoprim/sulfamethoxazole (110 µg); CIP, ciprofloxacin (5 µg); and AZM, azithromycin (10 µg). R, resistance; S, susceptibility; I, moderate susceptibility.

**Table 5:** Antibacterial activity of autochthonous lactic acid bacteria (LAB) isolates against three foodborne pathogens.

LAB Isolates	Inhibition Zone (mm)		
	*E. coli* ATCC 43895	*S. Enteritidis* ATCC 13076	*S. aureus* ATCC 25923
*P. pentosaceus* TAA01	16.0 ± 0.7	16.3 ± 0.8	12.7 ± 0.5
*L. curvatus* TAA04	12.0 ± 0.7	13.0 ± 1.0	ND
*L. mesenteroides* TDB19	ND	ND	9.7 ± 0.5
*L. curvatus* TDB21	11.0 ± 1.4	9.7 ± 0.6	13.5 ± 0.7
*L. mesenteroides* TDB22	14.0 ± 0.7	17.0 ± 1.0	14.0 ± 1.0
*L. curvatus* TDM40	11.3 ± 1.2	9.0 ± 1.0	ND
*L. plantarum* TDM41	14.7 ± 1.1	16.0 ± 1.0	14.0 ± 1.7

Data represent the mean ± standard deviation of three independent experiments. ND, not detected.

## Data Availability

The original contributions presented in the study are included in the article/supplementary material, further inquiries can be directed to the corresponding author/s.

## Supplementary Materials

The following supporting information can be downloaded at: https://www.mdpi.com/article/10.3390/foods13040575/s1. Table S1: Antibiotic sensitivity interpretative standard table.
