# Supplementary material for: Characterization of Autochthonous Lactic Acid Bacteria Isolated from a Traditional Ethiopian Beverage, Tella
Source: Foods. 2024 Feb 14;13(4):575. doi: 10.3390/foods13040575 (PMC10888401; doi:10.3390/foods13040575)
Supplement: Supplementary file 1 [file foods-13-00575-s001.zip › foods-2843694-supplementary.pdf]

Supplementary Table S1: Antibiotic sensitivity interpretative standard table

Class	Antibiotic	Disc concentration (μg)	Interpretative zone diameters (mm) [1]		
			R	I	S
ß-lactam	Ampicillin	10	≤13	13−15	≥15
Amino glycoside	Gentamicin	10	≤13	13−14	≥14
Amino glycoside	Kanamycin	30	≤14	14−17	≥17
Amino glycoside	Streptomycin	10	≤12	12−14	≥14
Macrolides	Erythromycin	15	≤14	14−22	≥22
Tetracyclines	Tetracycline	30	≤15	15−18	≥18
Phenicols	Chloramphenicol	30	≤14	14−17	≥17
ß-lactam	Penicillin G	10	≤13	13−17	≥17
sulfonamides	Sulfamethoxazole	110	≤13	13−17	≥17
Quinolones	Ciprofloxacin	5	≤13	13−17	≥17
Macrolides	Azithromycin	10	≤13	13−18	≥18

[1] The interpretation standard referred to the CLSI criteria (Haghshenas et al., 2014; Won et al., 2020)

S, Susceptible; I, Intermediate; R, Resistant
